# Rehabilitation and Physiotherapy Action Strategy for an Acute Case of Lateral Medullary Syndrome: A Case Report

**DOI:** 10.7759/cureus.70242

**Published:** 2024-09-26

**Authors:** Gauri Kariya, Rajat M Singh, Taj Afreen Sheikh

**Affiliations:** 1 Cardiovascular and Respiratory Physiotherapy, Ravi Nair Physiotherapy College, Datta Meghe Institute of Higher Education and Research, Wardha, IND

**Keywords:** bronchial hygiene, cerebro-vascular accident (stroke), chest physiotherapy, early ct brain, lateral medullary syndrome (wallenberg syndrome), mri brain

## Abstract

Lateral medullary syndrome (LMS) may result from a failure in either the vertebral artery or the posterior inferior cerebellar artery. Stroke is the most common cause of LMS. To achieve bronchial hygiene and improve the patient's condition, chest physiotherapy was initiated due to his confined condition during the acute stage. As a result, a four-week physiotherapy program was established and administered twice daily to the patient, with noticeable results. A physical examination revealed that the patient exhibited tachycardia, dyspnea, intracranial nerve palsies on the left side, thermoanesthesia on the right, horizontal nystagmus, and ptosis and miosis in the left eye associated with Horner syndrome. After receiving appropriate conservative care, the patient was discharged from the hospital with the fewest possible disabilities.

## Introduction

A cerebral vascular accident (CVA) is one of the most common causes of upper motor neuron lesions, especially as we age. The most prevalent kind of hemiplegia results from an internal capsule CVA. An infarction in the lateral medulla, which is located posterior to the inferior olivary nucleus, is the primary cause of Wallenberg syndrome, posterior inferior cerebellar artery (PICA) syndrome, and lateral medullary syndrome (LMS) [1]. In LMS, the V, IX, and X cranial nerves are involved, which have some of the most prominent neurological symptoms. LMS is frequently caused by a vascular event in the lateral medulla oblongata, affecting either the PICA or the vertebral artery (VA) [2]. The condition known as Wallenberg syndrome, or LMS, is named for the renowned Jewish neurologist and neuroanatomist Adolf Wallenberg (1862-1949), who was employed in Germany [3,4]. LMS and PICA are very infrequent causes of stroke, often resulting from emboli or thrombosis in the PICA or VA [5,6].

This syndrome produces ipsilateral Horner syndrome, loss of pain and temperature sensation in the face, weakness of the palate, pharynx, and vocal cords, and cerebellar ataxia. Contralateral to the lesion, there is hemibody loss of pain and temperature sensation.

It is the clinical manifestation resulting from occlusion of the PICA, one of its branches, or the VA, in which the lateral part of the medulla oblongata infarcts, resulting in a typical pattern. The most commonly affected artery is PICA, specifically the lateral medullary segment.

## Case presentation

Patient information

A 39-year-old male presented with complaints of recurrent episodes of vomiting and headache, which were sudden in onset, and slurred speech for seven days. Furthermore, the patient complained of weakness in the left side of the facial muscles, as well as photophobia, which was aggravated by activities and relieved by rest. Post-assessment, further blood and urine tests were done, and they were all normal. Afterward, magnetic resonance imaging (MRI) of the brain was performed, which suggested a signal lesion involving the right corona radiata, internal capsule, and lentiform nucleus. A further computed tomography (CT) scan of the brain revealed acute infarction in the medulla on the left and in the left corona radiata, suggesting small ischemic disease. He was shifted to the Intensive Care Unit (ICU), and, along with medical management, physiotherapy interventions were provided. The patient was hemodynamically stable. Post-auscultation, air entry was reduced in the middle and lower zones with a wheezing sound. The sensory examination revealed no sensory impairments.

Motor examination

Informed consent was obtained from the patient. The patient was seen in a supine position with the head elevated to 30 degrees. According to the Modified Ashworth Scale, there was no increase in muscle tone. Table 1 below provides voluntary control grading.

**Table 1:** Voluntary control grading score

Extremities	Right	Left
Upper limb	Grade 0	Grade 0
Lower limb	Grade 1	Grade 1

Investigations

**Figure 1:**
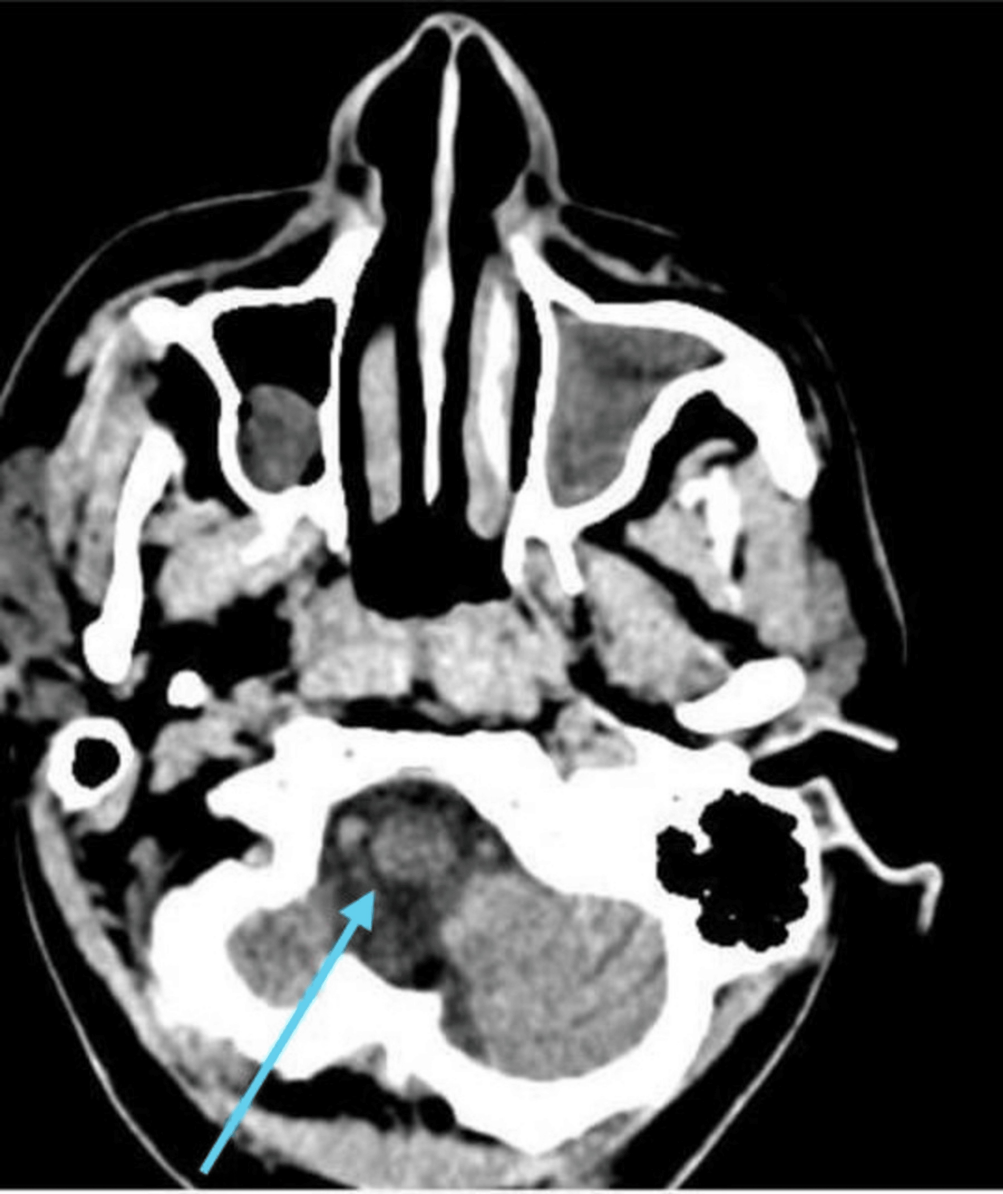
MRI of the brain The blue arrow shows a portion of the chronic infarct. MRI: Magnetic resonance imaging

The patient underwent clinical and radiological examinations. Figure 1 shows an MRI of the brain, indicating acute infarction in the medulla on the left and in the left corona radiata, suggesting small ischemic disease.

Therapeutic intervention

**Figure 2:**
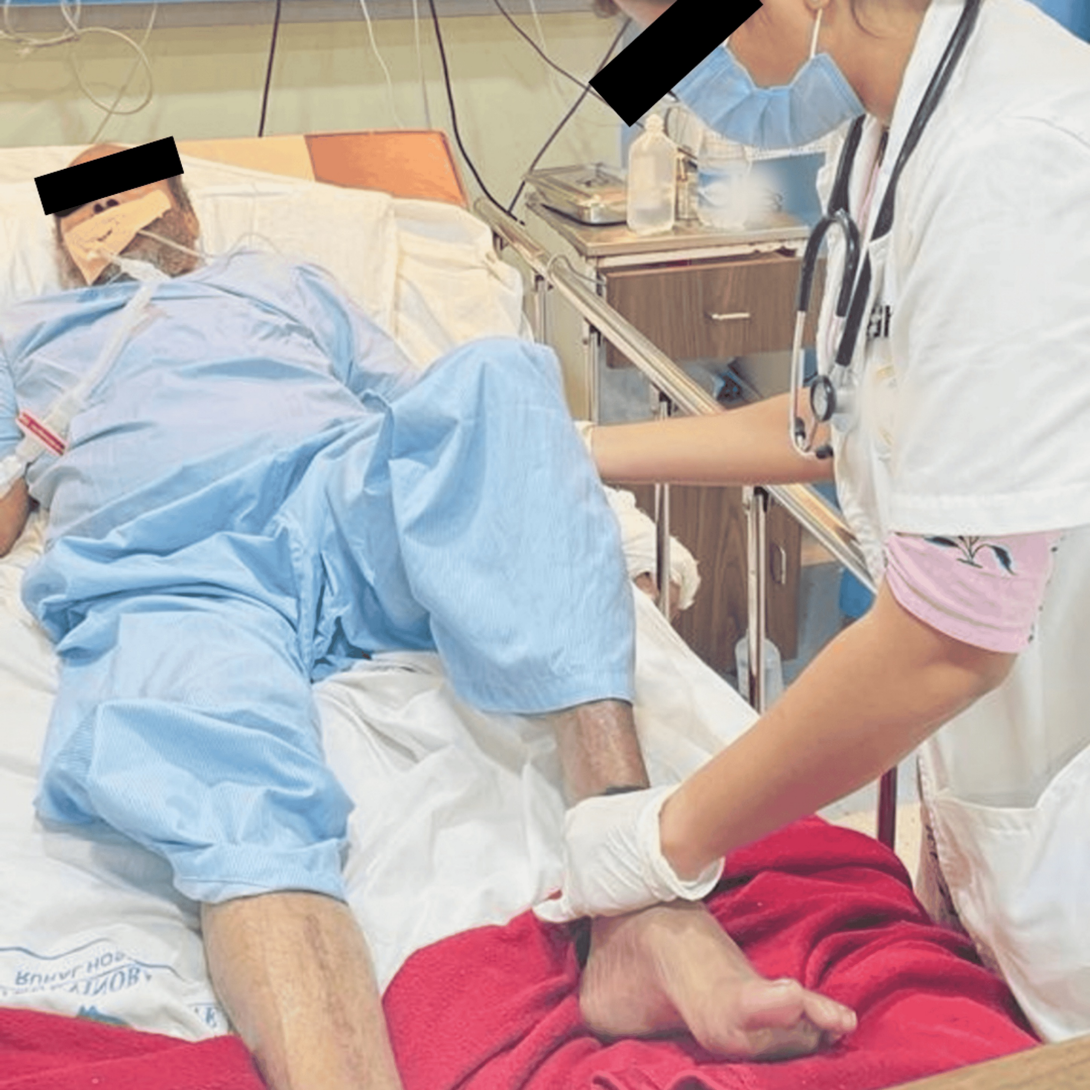
Therapist performing passive movements

**Figure 3:**
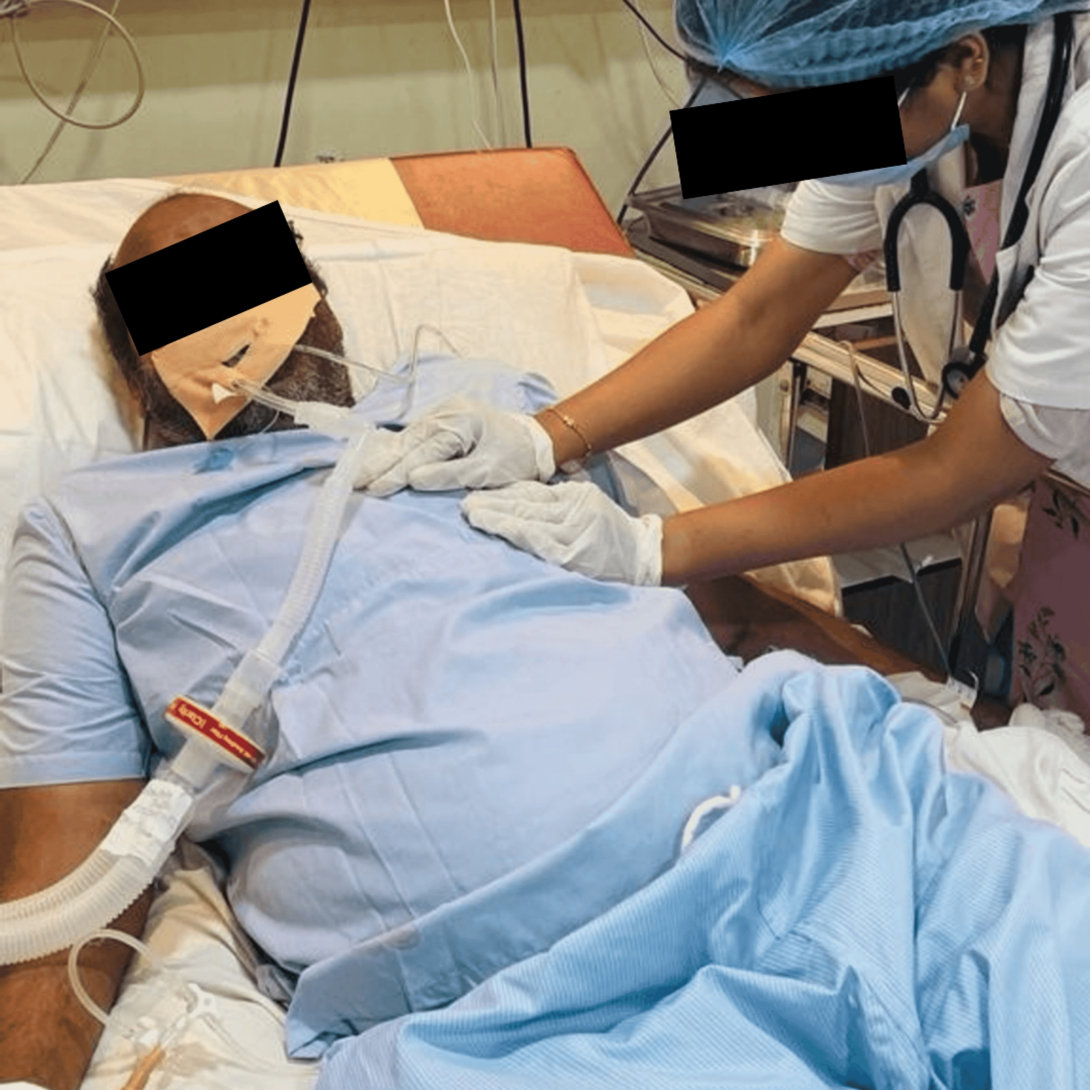
Therapist performing percussion

The patient's recovery was facilitated through the implementation of a collaborative approach involving multiple disciplines. This approach played a crucial role in ensuring a swift recuperation. The primary objective during the initial stage of treatment was to enhance and maintain bronchial hygiene, thereby minimizing the risk of complications. At this stage, the patient was unable to expectorate on their own. The results of pre-treatment assessments can be found in Table 2. Physiotherapy interventions were initiated promptly upon the patient's admission to the ICU. Initially, counseling to the patient's family was provided about the patient's condition and the physiotherapy interventions. Once the patient's condition stabilized, comprehensive counseling was given, as outlined in Table 3 and depicted in Figures 2-3. Table 2 shows the pre-treatment scores of scales, whereas Table 3 shows the treatment protocols for the patient.

**Table 2:** Pre-treatment score of scales NPRS: Numerical pain rating scale; WHO: World Health Organization; ICU: Intensive care unit; GCS: Glasgow coma scale; FiO2: Fraction of inspired oxygen; CPAP: Continuous positive airway pressure

Scales	Scores
NPRS (on movement)	7/10
WHO quality of life-physical performance	37/100
ICU mobility scale	0
Richmond agitation and sedation scale	-1
GCS	12
Oxygen support	60% FiO_2_ on CPAP mode

**Table 3:** Treatment protocol of the patient ROM: Range of motion, PNF: Proprioceptive neuromuscular facilitation

Problem list	Goals	Intervention
Unaware about the condition	Awareness about the condition and treatment protocol	Counseling about the condition and the treatment protocol at the beginning of the treatment
Secretion retention	To remove secretions by mobilizing secretions from the periphery to the central. For airway clearance and bronchial hygiene	Chest vibration on anterior aspect bilaterally on upper and lower zones
Bronchospasm	To facilitate ventilation	Nebulization
Decreased chest expansion	To increase lung volume and increase lung capacity	Positive end-expiratory pressure
Immobility	Bed transitioning under observation to prevent bed sores and other complications of immobility	Positioning of the patient should be done on both sides under the supervision of the therapist
Accumulation of secretion in the airways	Clearing lung fields before and after physiotherapy treatment	Suctioning should be done when the patient is not able to expectorate actively and as soon as the patient gets stable does it actively, an active cycle of breathing techniques should be given (as a futuristic goal)
Decreased ROM and strength	Improve ROM and strength	PNF D1 flexion and extension hold and relax technique passive
Decreased pelvic motions	Facilitate pelvic motions and strengthen low back and hip extensors which will help to enhance motor control of lumbo-pelvic region	Pelvic bridging exercises with 30-sec hold

Follow-up

Later, after a month of pulmonary rehabilitation, the patient was weaned off from the continuous positive airway pressure (CPAP) mode of the ventilator to an oxygen mask. The patient was able to perform activities of daily living gradually, but supportive aid was required. Sessions of physiotherapy treatment were conducted twice a day in the ICU for four weeks. After four weeks of medical treatment, along with a physiotherapy approach, the patient showed improvement in all the problem areas mentioned above, which helped enable early ambulation (Table 4).

**Table 4:** Post-treatment scores of scales NPRS: Numerical pain rating scale; WHO: World Health Organization, ICU: Intensive care unit, L: Liter, GCS: Glasgow coma scale

Scales	Scores
NPRS (on movement) [7]	5/10
WHO quality of life-physical performance [8]	59/100
ICU mobility scale [9]	3
Richmond agitation and sedation scale [10]	0
GCS [11]	15
Oxygen support	5 L of O_2_ via face mask

## Discussion

This study has demonstrated that an infarct on the lateral medulla is the primary cause of Wallenberg syndrome, sometimes referred to as LMS [12]. The superior, middle, and inferior medullary arteries, which also comprise the VA and the PICA, were shown to be the strongest arteries linked to this condition [13]. Classic LMS signs and symptoms include ipsilateral Horner syndrome, ipsilateral cerebellar indications, and hemisensory impairment over the ipsilateral face and contralateral body. He said that conservative therapy, combined with symptomatic medication and physiotherapy, leads to early recovery [14]. A rare kind of stroke is called Wallenberg syndrome or LMS [15].

Caplan et al. (1986) discovered that the most common neurological symptoms and signs of LMS in individuals who had them were gait ataxia (88%), nystagmus (71%), nausea and vomiting (65%), dysphagia (62%), hoarseness (41%), and vertigo and dizziness (88%), as well as Horner's sign (88%) [16]. Four bilateral trigeminal patterns, 10 contralateral trigeminal patterns, and 11 crossover patterns (ipsilateral trigeminal-contralateral hemi body and limb) were among the sensory manifestations.

The participation of either the ascending secondary trigeminal tract, the descending trigeminal tract, or both tracts was probably the source of each of these patterns. There were two examples of isolated trigeminal sensory abnormalities and six cases of isolated hemibody and limb sensory involvement [17,18]. Due to the concurrent involvement of the ascending secondary trigeminal fibers positioned medial-ventrally, bilateral or contralateral trigeminal sensory involvement was present in large MRI lesions [19].

Day et al. (2014) state that understanding the symptoms and indicators of LMS is essential for patient care. In order to rule out different diagnoses and treatments for acute stroke that are contraindicated, affected patients should obtain immediate neuroimaging. To rule out vascular disease, neurovascular imaging should be acquired whenever possible [20].

## Conclusions

This case report concludes by emphasizing the vital significance that a customized rehabilitation and physiotherapy action plan plays in the treatment of an acute LMS patient. The patient's functional abilities and quality of life were significantly improved by a comprehensive strategy that included targeted exercises, coordination training, and patient education. This instance emphasizes how crucial early intervention and a multidisciplinary approach are in helping patients with severe neurological diseases recover as much as possible. The efficacy of particular rehabilitation regimens should be further examined in future research to improve the comprehension and management of cases similar to this one.
